# Supplementary material for: Environmental Barriers and Functional Outcomes in Patients with Schizophrenia in Taiwan: The Capacity-Performance Discrepancy
Source: Int J Environ Res Public Health. 2021 Dec 28;19(1):315. doi: 10.3390/ijerph19010315 (PMC8751039; doi:10.3390/ijerph19010315)
Supplement: Supplementary file 1 [file ijerph-19-00315-s001.zip › ijerph-1489233-supplementary.pdf]

Supplementary Materials

Environmental barriers

The environmental barriers in the Taiwan Data Bank of Persons with Disability and Care Needs from the Social and Family Affairs Administration, Ministry of Health and Welfare, Taiwan (R.O.C.) included e1 product and technology subcategories e110 (products or substances for personal consumption), e115 (products and technology for personal use in daily living), e120 (products and technology for personal indoor and outdoor mobility and transportation), e125 (products and technology for communication), e130 (products and technology for education), and e165 (assets) accessibility. Based on the WHO online browser [1], the questions about accessibility for these categories were as follows: "e110: Did you have any difficulty in getting any natural or human-made object or substance gathered, processed, or manufactured for ingestion including food, drink and drugs?", "e115: Did you have any difficulty in getting any equipment, products, and technologies used by people in daily activities, including general and assistive products and technology for personal use?", "e120: Did you have any difficulty in getting any equipment, product,s and technologies used by people in activities of moving inside and outside buildings, including general and assistive products and technology for personal indoor and outdoor mobility and transportation?", "e125: Did you have any difficulty in getting any equipment, products, and technologies used by people in activities of sending and receiving information, including those adapted or specially designed, located in, on or near the person using them, including general and assistive products and technology for communication?", "e130: Did you have any difficulty in getting any equipment, products, processes, methods, and technology used for acquisition of knowledge, expertise, or skill, including those adapted or specially designed, including those adapted or specially designed, located in, on or near the person using them, including general and assistive products and technology for education?", and "e165: Did you have any difficulty in getting any products or objects of economic exchange such as money, goods, property, and other valuables that an individual owns or of which he or she has rights of use, including those adapted or specially designed, located in, on or near the person using them, including tangible and intangible products and goods, financial assets?"

References:

1. (WHO), W.H.O. *ICF browser*. 2013 [cited 2021 July 15]; Available from: https://apps.who.int/classifications/icfbrowser/.

Table S1. Logistic regression analysis of potential risk factors associated with having chapter e1 environmental accessibility barriers. ***p<0.001, **p<0.01, *p<0.05.

Environmental barriers	e110	e115	e120	e125	e130	e165
C statistic (95% CI)	0.765*** (0.739–0.792)	0.761*** (0.737–0.785)	0.722*** (0.700–0.743)	0.721*** (0.694–0.748)	0.682*** (0.655–0.708)	0.664*** (0.644–0.684)
Variables						
Severe schizophrenia (ref: Moderate schizophrenia)	0.88 (0.67–1.14)	0.92 (0.73–1.18)	1.03 (0.84–1.26)	1.29* (1.00–1.67)	0.99 (0.78–1.25)	1.07 (0.90–1.27)
Performance score	1.04*** (1.04–1.05)	1.04*** (1.04–1.05)	1.03*** (1.03–1.04)	1.03*** (1.02–1.03)	1.03*** (1.02–1.03)	1.03*** (1.02–1.03)
Elderly (ref: 18-64 years)	0.89 (0.66–1.19)	0.81 (0.61–1.07)	0.92 (0.72–1.16)	1.06 (0.80–1.42)	0.71* (0.53–0.95)	0.65*** (0.52–0.81)
Female (ref: male)	1.17 (0.92–1.49)	1.02 (0.82–1.27)	1.07 (0.89–1.29)	0.86 (0.68–1.09)	0.87 (0.70–1.08)	0.97 (0.83–1.13)
No major caregiver (ref: Yes)	1.07 (0.74–1.54)	0.92 (0.66–1.27)	0.79 (0.60–1.04)	0.86 (0.61–1.23)	0.83 (0.61–1.14)	0.89 (0.71–1.12)
Lower educational level (ref: Above primary school)	1.13 (0.89–1.44)	1.16 (0.93–1.45)	1.22* (1.01–1.47)	1.24 (0.98–1.57)	1.08 (0.87–1.35)	1.02 (0.86–1.19)
Institution (ref: Community dwelling)	0.94 (0.65–1.36)	0.88 (0.63–1.24)	0.91 (0.69–1.20)	0.94 (0.66–1.35)	0.89 (0.65–1.23)	0.79* (0.63–0.98)
Urbanization level						
Suburban (ref: Rural)	1.31 (0.99–1.72)	1.33* (1.03–1.71)	1.07 (0.86–1.33)	1.82*** (1.39–2.40)	1.38* (1.08–1.76)	1.21* (1.00–1.45)
Urban (ref: Rural)	1.26 (0.93–1.69)	1.41* (1.07–1.84)	1.14 (0.90–1.43)	1.65*** (1.23–2.22)	1.09 (0.83–1.43)	1.31*** (1.08–1.59)
Unemployment (ref: Employment)	1.64 (0.59–4.56)	1.46 (0.63–3.40)	2.27* (1.04–4.95)	1.47 (0.59–3.69)	1.14 (0.59–2.24)	1.29 (0.82–2.02)
Middle-low –low family economic status (ref: General)	1.34* (1.05–1.70)	1.03 (0.82–1.28)	0.93 (0.77–1.13)	0.95 (0.74–1.20)	1.16 (0.93–1.44)	0.96 (0.81–1.12)

CI: confidence interval.

Table S2. The area under curve (AUC), cut-off value, sensitivity, and specificity of ROC analyses

	Moderate schizophrenia (n = 2497)	Severe schizophrenia (n = 1385)
AUC (95% CI)		
e110	0.786 (0.749–0.824)	0.703 (0.661–0.746)
e115	0.774 (0.742–0.807)	0.676 (0.641–0.712)
e120	0.733 (0.705–0.762)	0.673 (0.640–0.706)
Cut-off value		
e110	40	55
e115	34	55
e120	34	55
Sensitivity		
e110	0.713	0.694
e115	0.712	0.607
e120	0.708	0.592
Specificity		
e110	0.752	0.653
e115	0.677	0.666
e120	0.675	0.673

ROC analyses to classify patients with schizophrenia according to the severity of disability experiencing an accessibility barrier to ICF category e110, e115, and e120 by utilizing the standardized summary index scores of performance. CI: confidence interval.

Table S3. The comparison of the overall summary index (SI) of performance in patients with moderate schizophrenia without e110, e115, or e120 accessibility barrier and those without any e110, e115, and e120 accessibility barriers

	**SI of performance (mean±SD)**
without e110 accessibility barrier (n=2323)	29.4±18.7
without e110, e115, and e120 accessibility barriers (n=2096)	28.3±18.4
p value	0.05
without e115 accessibility barrier (n=2282)	29.1±18.6
without e110, e115, and e120 accessibility barriers (n=2096)	28.3±18.4

p value	0.12
without e120 accessibility barrier (n=2178)	28.8±18.7
without e110, e115, and e120 accessibility barriers (n=2096)	28.3±18.4
p value	0.31

Table S4. The comparison of the overall summary index (SI) of performance in patients with severe schizophrenia without e110, e115, or e120 accessibility barrier and those without any e110, e115, and e120 accessibility barriers

	**SI of performance (mean±SD)**
without e110 accessibility barrier (n=1215)	45.8±23.6
without e110, e115, and e120 accessibility barriers (n=1046)	44.7±23.3
p value	0.26
without e115 accessibility barrier (n=1182)	45.4±23.6
without e110, e115, and e120 accessibility barriers (n=1046)	44.7±23.3
p value	0.49
without e120 accessibility barrier (n=1110)	44.8±23.4
without e110, e115, and e120 accessibility barriers (n=1046)	44.7±23.3
p value	0.92

Table S5. Demographics of the study population and WHODAS 2.0 evaluation results using multiple imputation

	**Moderate schizophrenia (n=2845)**	**Severe schizophrenia (n=1594)**	***p***	**Statistics (statistical tests)**	**Degree of freedom (df)**
Female (n, %)	1,330 (46.8)	737 (46.2)	0.743	0.108 (chi-squared test)	1
Age (years old, mean [SD])	48.6 (14.3)	55.9 (13.4)	<0.001	17.142 (t-test)	3483.7
Education			<0.001	89.721 (chi-squared test)	1
>Primary	1,902 (66.9)	836 (52.4)			
≤Primary	943 (33.2)	758 (47.6)			
Residence			<0.001	373.491 (chi-squared test)	1
Community	1,185 (41.7)	216 (14.5)			
Institution	1,660 (58.4)	1,378 (86.5)			
Primary caregiver			<0.001	85.178 (chi-squared test)	1

Yes	892 (31.4)	296 (18.6)			
No	1,953 (68.6)	1,298 (81.4)			
Urbanization level			0.005	10.490 (chi-squared test)	2
Rural	639 (22.5)	427 (26.8)			
Suburban	793 (27.9)	421 (26.4)			
Urban	1,413 (49.7)	746 (46.5)			
Work status			<0.001	82.982 (chi-squared test)	1
Employment	172 (6.1)	7 (0.4)			
Unemployment	2,673 (94.0)	1,587 (99.6)			
Family economic status			<0.001	53.286 (chi-squared test)	1
General	1,650 (58.0)	743 (46.6)			
Middle low–low	1,195 (42.0)	851 (53.4)			
WHODAS 2.0 (mean [SD])					
Cognition (domain 1)					
Capacity	38.4 (25.5)	59.9 (28.6)	<0.001	24.918 (t-test)	2991
Performance	36.3 (24.6)	57.5 (28.5)	<0.001	24.847 (t-test)	2913
Mobility (domain 2)					
Capacity	20.4 (28.6)	39.9 (36.8)	<0.001	18.343 (t-test)	2684.5
Performance	17.7 (25.2)	34.7 (33.2)	<0.001	17.802 (t-test)	2633.6
Self-care (domain 3)					
Capacity	19.0 (25.8)	41.5 (34.3)	<0.001	22.744 (t-test)	2614.1
Performance	14.7 (21.2)	31.1 (21.2)	<0.001	19.009 (t-test)	2464.7
Getting along (domain 4)					
Capacity	40.0 (25.6)	55.9 (28.8)	<0.001	18.357 (t-test)	2985.4
Performance	39.1 (25.2)	54.4 (28.7)	<0.001	17.794 (t-test)	2956.3
Life activities (domain 5-1)					
Capacity	44.2 (33.2)	65.3 (37.4)	<0.001	18.793 (t-test)	2985.1
Performance	40.6 (32.6)	60.3 (38.5)	<0.001	17.282 (t-test)	2873.7
Social participation (domain 6)					
Capacity	36.5 (23.3)	48.5 (26.6)	<0.001	13.871 (t-test)	2913.4
Performance	34.5 (22.1)	45.1 (25.7)	<0.001	15.143 (t-test)	2951.3
Overall summary index (SI)					
Capacity	33.5 (21.7)	51.5 (25.3)	<0.001	22.910 (t-test)	2825.1
Performance	31.1 (20.0)	47.3 (24.1)	<0.001	23.960 (t-test)	2899
Chapter e1	837 (29.4)	596 (37.4)	<0.001	29.686 (chi-squared test)	1
Category e110	202 (7.1)	172 (12.1)	<0.001	30.887 (chi-squared test)	1
Category e115	246 (8.7)	231 (14.5)	<0.001	36.392 (chi-squared test)	1

Category e120	363 (12.8)	326 (20.5)	<0.001	46.104 (chi-squared test)	1
Category e125	198 (7.0)	204 (12.8)	<0.001	42.283 (chi-squared test)	1
Category e130	264 (9.3)	207 (13.0)	<0.001	14.800 (chi-squared test)	1
Category e165	599 (21.1)	446 (28.0)	<0.001	27.222 (chi-squared test)	1

Table S6. The sociodemographic allocation and comparison of the patients with schizophrenia without and with accessibility barriers in the categories of chapter e1 products and technology with multiple imputation

**Parameters**	**e110 without accessibility barrier**	**e110 with accessibility barrier**	***p***	**e115 without accessibility barrier**	**e115 with accessibility barrier**	***p***	**e120 without accessibility barrier**	**e120 with accessibility barrier**	***p***
Total	4,045 (91.1)	394 (8.9)		3,962 (89.3)	477 (10.8)		3,750 (84.5)	689 (15.5)	
Age groups			<0.001			0.008			<0.001
18-64 years	3,359 (83.0)	298 (75.6)		3,285 (82.9)	372 (78.0)		3,125 (83.3)	532 (77.2)	
≥65 years	686 (17.0)	96 (24.4)		677 (17.1)	105 (22.0)		625 (16.7)	157 (22.8)	
Impairment			<0.001			<0.001			<0.001
Moderate	2,643 (65.3)	202 (51.3)		2,599 (65.6)	246 (51.6)		2,482 (66.2)	363 (52.7)	
Severe	1,402 (34.7)	192 (48.7)		1,363 (34.4)	231 (48.4)		1,268 (33.8)	326 (47.3)	
Sex			0.030			0.337			0.094
Male	2,182 (53.9)	190 (48.2)		2,127 (53.7)	245 (51.4)		2,024 (54.0)	348 (50.5)	
Female	1,863 (46.1)	204 (51.8)		1,835 (46.3)	232 (48.6)		1,726 (46.0)	341 (49.5)	
Primary caregiver			0.085			0.856			0.369
Yes	1,097 (27.1)	91 (23.1)		1,062 (26.8)	126 (26.4)		994 (26.5)	194 (28.2)	
No	2,948 (72.9)	303 (76.9)		2,900 (73.2)	351 (73.6)		2,756 (73.5)	495 (71.8)	
Education			<0.001			<0.001			<0.001
>Primary	2,533 (62.6)	205 (52.0)		2,482 (62.7)	256 (53.7)		2,364 (63.0)	374 (54.3)	
≤Primary	1,512 (37.4)	189 (48.0)		1,480 (37.4)	221 (46.3)		1,386 (37.0)	315 (45.7)	
Residence			0.003			0.032			0.045
Community	1,303 (32.2)	98 (24.9)		1,271 (32.1)	130 (27.2)		1,206 (32.2)	195 (28.3)	
Institution	2,742 (67.8)	296 (75.1)		2,691 (67.9)	347 (72.8)		2,544 (67.8)	494 (71.7)	

Urbanization level			0.031			0.008			0.272
Rural	963 (23.8)	103 (26.1)		934 (23.6)	132 (27.7)		889 (23.7)	177 (25.7)	
Suburban	1,090 (27.0)	124 (31.5)		1,069 (27.0)	145 (30.4)		1,018 (27.2)	196 (28.5)	
Urban	1,992 (49.3)	167 (42.4)		1,959 (49.4)	200 (41.9)		1,843 (49.2)	316 (45.9)	
Work status			0.004			0.003			<0.001
Employed	174 (4.3)	5 (1.3)		172 (4.3)	7 (1.5)		169 (4.5)	10 (1.4)	
Unemployed	3,871 (95.7)	389 (98.7)		3,790 (95.7)	470 (98.5)		3,581 (95.5)	679 (98.6)	
Family economic status			<0.001			0.063			0.593
General	2,212 (54.7)	181 (45.9)		2,155 (54.4)	238 (49.9)		2,028 (54.1)	365 (53.0)	
Middle low–Low	1,833 (45.3)	213 (54.1)		1,807 (45.6)	239 (50.1)		1,722 (45.)	324 (47.0)	

Table S7. Comparison of relative difference (RD, capacity-performance discrepancy) of the summary index (SI) between schizophrenia patients with and without accessibility barriers to the categories in chapter e1 products and technology with multiple imputation, including environmental categories e110 for personal consumption, e115 for personal usage in activities of daily living, and e120 for personal outdoor and indoor mobility and transportation, stratified by patients with moderate schizophrenia (n = 202 in accessibility of e110 with barrier, n = 246 in accessibility of e115 with barrier, n = 363 in accessibility of e120 with barrier) and severe schizophrenia (n = 192 in accessibility of e110 with barrier, n = 231 in accessibility of e115 with barrier, n = 326 in accessibility of e120 with barrier).

	**Moderate schizophrenia (n=2845)**	**Severe schizophrenia (n=1594)**
Accessibility of e110		
with barrier	0** (0–10.28)	0 (0–9.14)
without barrier	0** (0–4.26)	0 (0–10.31)
Accessibility of e115		
with barrier	0** (0–10.47)	0 (0–8.44)
without barrier	0** (0–4.29)	0 (0–10.46)
Accessibility of e120		
with barrier	0** (0–11.88)	0 (0–9.60)
without barrier	0** (0–3.34)	0 (0–10.27)

Table S8. The area under the curve (AUC), cut-off value, sensitivity, and specificity of ROC analyses with multiple imputation

	**Moderate schizophrenia (n=2845)**	**Severe schizophrenia (n=1594)**
AUC (95% CI)		
e110	0.778 (0.742–0.814)	0.692 (0.651–0.732)
e115	0.774 (0.743–0.804)	0.695 (0.659–0.731)
e120	0.733 (0.706–0.760)	0.673 (0.640–0.706)
Cut-off value		
e110	40	52
e115	35	52
e120	34	56
Sensitivity		
e110	0.713	0.719
e115	0.724	0.706
e120	0.708	0.580
Specificity		
e110	0.750	0.608
e115	0.686	0.616
e120	0.675	0.689

Table S9. The area under curve (AUC), cut-off value, sensitivity, and specificity of ROC analyses after stratification by sex (male and female) and age (18-64 years and ≥65 years)

	Male				Female			
	18-64		≥65		18-64		≥65	
	Moderate schizophrenia (n=1192)	Severe schizophrenia (n=601)	Moderate schizophrenia (n=126)	Severe schizophrenia (n=147)	Moderate schizophrenia (n=981)	Severe schizophrenia (n=420)	Moderate schizophrenia (n=198)	Severe schizophrenia (n=217)
AUC (95% CI)								
e110	0.820 (0.767–0.873)	0.708 (0.641–0.776)	0.820 (0.664–0.976)	0.586 (0.427–0.745)	0.759 (0.696–0.821)	0.736 (0.658–0.813)	0.729 (0.612–0.845)	0.686 (0.594–0.777)
e115	0.796 (0.747–0.846)	0.693 (0.636–0.751)	0.647 (0.442–0.853)	0.658 (0.537–0.778)	0.787 (0.738–0.836)	0.733 (0.661–0.805)	0.650 (0.541–0.759)	0.711 (0.624–0.798)
e120	0.750 (0.707–0.793)	0.666 (0.611–0.721)	0.697 (0.557–0.838)	0.572 (0.456–0.689)	0.734 (0.689–0.780)	0.718 (0.655–0.782)	0.607 (0.515–0.670)	0.670 (0.584–0.756)
Cut-off value								
e110	40	52	68	57	39	51	49	63
e115	35	52	35	57	30	55	49	64
e120	34	56	35	57	33	65	46	63
Sensitivity								
e110	0.750	0.746	0.714	0.706	0.657	0.761	0.810	0.700
e115	0.753	0.682	0.636	0.833	0.813	0.741	0.667	0.667
e120	0.697	0.535	0.765	0.645	0.697	0.517	0.579	0.623
Specificity								
e110	0.792	0.657	0.908	0.523	0.764	0.626	0.644	0.661
e115	0.729	0.663	0.530	0.543	0.618	0.635	0.637	0.691
e120	0.707	0.739	0.560	0.534	0.696	0.835	0.613	0.665

Table S10. Comparison of relative difference (RD, capacity-performance discrepancy) of the summary index (SI) between schizophrenia patients with and without accessibility barriers to the categories in chapter e1 products and technology, including environmental categories e110 for personal consumption, e115 for personal usage in activities of daily living, and e120 for personal outdoor and indoor mobility and transportation, stratified by patients with moderate schizophrenia and severe schizophrenia and sex and age.

			**Moderate schizophrenia**		**Severe schizophrenia**	
			N	**RD**	N	**RD**
Accessibility of e110						
Male	18-64	with barrier	76	0** (0–8.79)	67	0 (0–10.01)
		without barrier	1116	0** (0–0)	534	0 (0–8.01)
	65+	with barrier	7	0 (0–7.05)	17	0 (0–2.44)

		without barrier	119	0 (0–11.93)	130	0 (0–14.33)
Female	18-64	with barrier	70	0** (0–12.32)	46	0 (0–8.54)
		without barrier	911	0** (0–2.51)	374	0 (0–10.89)
	65+	with barrier	21	0 (0–5.67)	40	4.34 (0–12.49)
		without barrier	177	3.04 (0–16.70)	177	5.72 (0–15.03)
Accessibility of e115						
Male	18-64	with barrier	97	0*** (0–9.43)	88	0 (0–10.32)
		without barrier	1095	0*** (0–0)	513	0 (0–8.01)
	65+	with barrier	11	0 (0–11.93)	18	0.62 (0–4.69)
		without barrier	115	0 (0–11.65)	129	0 (0–13.23)
Female	18-64	with barrier	80	0* (0–9.35)	58	1.12 (0–9.60)
		without barrier	901	0* (0–2.86)	362	0 (0–10.83)
	65+	with barrier	27	0 (0–9.53)	39	4.06 (0–11.12)
		without barrier	171	3.04 (0–16.70)	178	5.75 (0–15.82)
Accessibility of e120						
Male	18-64	with barrier	142	0*** (0–11.50)	114	0 (0–10.01)
		without barrier	1050	0*** (0–0)	487	0 (0–7.86)
	65+	with barrier	17	0***(0–11.93)	31	0 (0–5.34)
		without barrier	109	0***(0–11.34)	116	0 (0–14.40)
Female	18-64	with barrier	122	0 (0–9.70)	87	0 (0–7.25)
		without barrier	859	0 (0–1.52)	333	0 (0–11.12)
	65+	with barrier	38	0***(0–15.41)	53	5.78 (0–13.20)
		without barrier	160	2.84***(0–15.29)	164	5.33 (0–15.06)

Table S11. The factors with substantial difference in relative difference (RD, capacity-performance discrepancy) in patients with severe schizophrenia and e120 accessibility barrier (n = 285). Median (interquartile range [IQR]).

Severe schizophrenia and e120 accessibility barrier (+)	Median (IQR)	Median (IQR)	*P*†
18-64 years vs ≥65 years	0 (0–8.63)	3.50 (0–9.87)	0.048*
Male vs female	0 (0–8.34)	1.14 (0–9.62)	0.254
Above primary school vs Lower education level	0 (0–9.85)	0.55 (0–8.91)	0.978
Major caregiver Yes vs No	4.81 (0–12.69)	0 (0–8.34)	0.033*
Community vs Institution	5.14 (0–12.69)	0 (0–8.39)	0.030*
Rural areas vs Suburban areas	1.11 (0–8.97)	0 (0–9.22)	0.787
Rural areas vs Urban areas	1.11 (0–8.97)	0 (0–9.60)	0.477
General vs Middle low –low family economic status	1.10 (0–9.85)	0 (0–8.44)	0.153

*$P < 0.05$.

†Mann–Whitney U test or Kruskal-Wallis test.

‡The results of the comparison between employment and unemployment are not shown because there was only one patient in the employed group.

STROBE Statement—Checklist of items that should be included in reports of ***cross-sectional studies***

	Item No	Recommendation	
**Title and abstract**	1	(*a*) Indicate the study's design with a commonly used term in the title or the abstract	**Page 1**
		(*b*) Provide in the abstract an informative and balanced summary of what was done and what was found	**Page 1**
**Introduction**			
Background/rationale	2	Explain the scientific background and rationale for the investigation being reported	**Page 1**
Objectives	3	State specific objectives, including any prespecified hypotheses	**Page 2**
**Methods**			
Study design	4	Present key elements of study design early in the paper	**Pages 3-4**
Setting	5	Describe the setting, locations, and relevant dates, including periods of recruitment, exposure, follow-up, and data collection	**Pages 3-4**
Participants	6	(*a*) Give the eligibility criteria, and the sources and methods of selection of participants	**Pages 3-4**
Variables	7	Clearly define all outcomes, exposures, predictors, potential confounders, and effect modifiers. Give diagnostic criteria, if applicable	**Pages 3-4**
Data sources/ measurement	8*	For each variable of interest, give sources of data and details of methods of assessment (measurement). Describe comparability of assessment methods if there is more than one group	**Pages 3-4**
Bias	9	Describe any efforts to address potential sources of bias	**Page 12**
Study size	10	Explain how the study size was arrived at	**Pages 4-5**
Quantitative variables	11	Explain how quantitative variables were handled in the analyses. If applicable, describe which groupings were chosen and why	**Pages 3-4**
Statistical methods	12	(*a*) Describe all statistical methods, including those used to control for confounding	**Pages 4-5**
		(*b*) Describe any methods used to examine subgroups and interactions	**Pages 4-5, 12**
		(*c*) Explain how missing data were addressed	**Page 12**
		(*d*) If applicable, describe analytical methods taking account of sampling strategy	**N/A**
		(*e*) Describe any sensitivity analyses	**Page 12**
**Results**			
Participants	13*	(a) Report numbers of individuals at each stage of study—eg numbers potentially eligible, examined for eligibility, confirmed eligible, included in the study, completing follow-up, and analysed	**Pages 6-7**
		(b) Give reasons for non-participation at each stage	**Page 6**
		(c) Consider use of a flow diagram	**Page 6**
Descriptive data	14*	(a) Give characteristics of study participants (eg demographic, clinical, social) and information on exposures and potential confounders	**Page 6**
		(b) Indicate number of participants with missing data for each variable of interest	**Page 6**
Outcome data	15*	Report numbers of outcome events or summary measures	**Pages 6-11**
Main results	16	(*a*) Give unadjusted estimates and, if applicable, confounder-adjusted estimates and their precision (eg, 95% confidence interval). Make clear which confounders were adjusted for and why they were included	**Pages 8, 11-12**
		(*b*) Report category boundaries when continuous variables were categorized	**Pages 8-10**

		(*c*) If relevant, consider translating estimates of relative risk into absolute risk for a meaningful time period	**N/A**
Other analyses	17	Report other analyses done—eg analyses of subgroups and interactions, and sensitivity analyses	**Page 12**
**Discussion**			
Key results	18	Summarise key results with reference to study objectives	**Pages 12-13**
Limitations	19	Discuss limitations of the study, taking into account sources of potential bias or imprecision. Discuss both direction and magnitude of any potential bias	**Page 14**
Interpretation	20	Give a cautious overall interpretation of results considering objectives, limitations, multiplicity of analyses, results from similar studies, and other relevant evidence	**Pages 12-14**
Generalisability	21	Discuss the generalisability (external validity) of the study results	**Page 14**
**Other information**			
Funding	22	Give the source of funding and the role of the funders for the present study and, if applicable, for the original study on which the present article is based	**Page 15**

*Give information separately for exposed and unexposed groups.

Note: An Explanation and Elaboration article discusses each checklist item and gives methodological background and published examples of transparent reporting. The STROBE checklist is best used in conjunction with this article (freely available on the Web sites of PLoS Medicine at http://www.plosmedicine.org/, Annals of Internal Medicine at http://www.annals.org/, and Epidemiology at http://www.epidem.com/). Information on the STROBE Initiative is available at www.strobe-statement.org.
